# Supplementary material for: Patterns of Intron Gain and Loss in Fungi
Source: PLoS Biol. 2004 Nov 30;2(12):e422. doi: 10.1371/journal.pbio.0020422 (PMC532390; doi:10.1371/journal.pbio.0020422)
Supplement: Table S1 — Also available at http://genes.mit.edu/NielsenEtAl/. (4.3 MB ZIP). [file pbio.0020422.st001.zip › NielsenEtAl/html/812.html]

AN6119.1.NCU01464.1.MG06906.1.FG01898.1


```
 CLUSTAL W (1.82) Multiple Sequence Alignments - Introns Inserted


Sequence 1: NCU01464.1	536 aa
Sequence 2: MG06906.1	567 aa
Sequence 3: FG01898.1	563 aa
Sequence 4: AN6119.1	573 aa
Alignment Length: 612 aa
Number Identitical Residues: 159 aa
Alignment Score (without introns) 8732


MG06906.1 	MHSRPYFTTPILLCLALALVFCP-TVPAAGVRLDHG------YASYQPPQAQANNGVFPF
NCU01464.1	--MRPSPESLSVLPLVIGVVLSPSTVDAAAFRHAGQPAGLIDTANSDDLYRGARGSALPG
FG01898.1 	--MKLQLLTASLLLLQASNSNINVNANAVGINPQRD----IPASRNTDSHAQSNTRLFPY
AN6119.1  	--MR-SFRSLHLLLLAASAITADAVPAGSSITPPPP-------VEPVSLLSSHSNPQRP-
          	   :    :  :* *  .         . ..                           * 

MG06906.1 	LTWLRNSAVEFIFGRPASAKPKSAPARGMG--AFNPRYSKETVLRFNVSSQEDENALADA
NCU01464.1	LTWLRDSISEIIFGRPSTKHSDSISPS-----VSLSQYRDDVVVRFNVIKPADQVVLAQA
FG01898.1 	LTKLRDKAIESIFGCKPTQDATPPPVHN----QLRAQYVKQLVLRFNVTTSHEEGALADA
AN6119.1  	WTRLRNWVIESIWGIPKSQTSRPTAHDYSAPLQGSTRYGSDVVLRFRVQGLDEAEALTEA
          	 * **:   * *:*   :  . . .    ..    .:* .: *:**.*    :  .*::*

MG06906.1 	ADRLFLDVWGFTRNHVDIRIHKND~VTPLLSLLPVSLRGNFSTLIPDLPSAVAATYNRRV
NCU01464.1	IKAKRLDVWASTPQFVDIRLEESQ~LRPLLRLIPSSMQKQWSVLIPDLEEAVRATFPTKG
FG01898.1 	AARLFLDVWAFTDDYVDIRLHADE~VRPLLSLLPKSLHTSHSILIPDLATAVYNSLPTGG
AN6119.1  	TNILFLDVWASTTEFVDIRLAKEV0T-------------AYVPLIDNLPEAISATYPARR
          	     ****. * :.****:  .                    ** :*  *:  :     

MG06906.1 	KDDTRFDPSSSKTLKPSLIDSAEEFDNVFFRDYQPLH~VIHQWMRLLESMFPKIVKLETI
NCU01464.1	ETTEYGDAAES-------LDAVYTKGNIFFHDYQPLS0VITSWMALMETMFPSIAKMESI
FG01898.1 	NP-AYTDPKTFAP---VVKIASSPVDNLFFQDYQSLP~VIMRWMRLLEAMFPSYVKYINV
AN6119.1  	SLGLNDQFMPS---------PSTRAPDLFFEDYQPLS~VITPWMQLMASMFSSHARMINV
          	.     :             .     ::**.***.*  **  ** *: :**.. .:  .:

MG06906.1 	GKSFEGREILALRVGNNLYAQDADKNKAPRKTVVINGGLHAREWIATATVNYLVWALITS
NCU01464.1	GDSYEGRPIHSFRIGD---HSGDNANNGTRKTILVTGGLHGREWISTSTVTYLLWSIITA
FG01898.1 	GKSYEGRDIPALRVGI----SHNVPNAPRRKTIMIMGGSHAREWISTSTVNYLAWSFITS
AN6119.1  	GVSYEGRQIPALRLGR------SSQPGESRPMILIVGGNHAREWISTSTVAYIAYNLMTR
          	* *:*** * ::*:*              *  ::: ** *.****:*:** *: : ::* 

MG06906.1 	YDQEPVLTRFLNNYTVVFIPVTNPDGYVYSWDSDRLWRKSRQDTPFNFCHGLDLNHAFDF
NCU01464.1	YGKESMVTKLLKHFDIIFIPVVNPDGYDYTWKTDRLWRKTRQQTKVSWCHGFDLDQAFGS
FG01898.1 	YGKERMITKLLDDFDLIFLPVANPDGFEYTWHVDRLWRKTRQQTNLQFCRGLDLDRAYGY
AN6119.1  	YGSSAAVTSLLQDYDWVLVPTVNPDGYVYTWDSDRLWRKNRQPTSFRFCPGIDLDRSWSF
          	*...  :* :*..:  :::*..****: *:*. ******.** * . :* *:**::::. 

MG06906.1 	EWNQD--LREPCSEDYGGQKPFEAVEARELSQWAKKQVEENHAQFVGFIDLHSYSQEILY
NCU01464.1	VSGQTNFQVDPCSESYGGDKPFQAVEATRLADWGRNQTEN-GVKFVGFLDLHSYSQQILF
FG01898.1 	EWDGSRVQRDPCSESYGGEKPFQAVEAVQIADWARNQTWNNNVRFVGLVDLHSYSQQILY
AN6119.1  	EWDGNQTQTNPCSENYAGEEPFEGVEAQQLAEWALNETEHNNARFVGFLDFHSYAQQILY
          	  .      :****.*.*::**:.*** .:::*. ::. .. .:***::*:***:*:**:

MG06906.1 	PFEYTCDPEPPNVENLEELAADLAKAIRLSSGEQYDVFSACEGAVL--SRHGGRRGRVHR
NCU01464.1	PYTSTCDEDPPNRENLEELGVGLAKAIRLSSGNFYTVSSACEGTTT--RQDAAN------
FG01898.1 	PFSFTCSVDPPNLENLEELAAGIAKSIRLSNGESYTVASACEGAVS--AREFSGGRYWSR
AN6119.1  	PYTYSCSSVPPTLESLEELGLGLAKVIRLTTHEIYDVTSACEGVVIPASGDKSAKSLFPV
          	*:  :*.  **. *.****. .:** ***:. : * * *****.. .:  . .       

MG06906.1 	MEPPGGSAMDWFYH~EMHARYSYQIKLRDRGSYGFLLPPDAIIPTGKEVFNALKFYADFL
NCU01464.1	-EATGGSAIDWVYH1ELYSRYSYQVKLRDTGIYGFLLPRSQIIPTGEEMLNALKYFGDFL
FG01898.1 	IESGGGSAVDWFYH~EMRAHYSYQIKLRDTGSYGFLLPKEHIVPTGEEIFNAMKYYGDYL
AN6119.1  	GGSSGGSGLDWFYH~QLRTRYAYQIKLRDRGSYGFLLPSEYIVPTGTEAFNAVLKLGQFL
          	  . ***.:**.** :: ::*:**:**** * ****** . *:*** * :**:   .::*

MG06906.1 	GGNN-----GIERVSSSRPFGPVTAQKP------------EDVDAEIDEMAELKKRRRKR
NCU01464.1	LGNN-----GIESISKAKER---TSHDK------------DRVDLK--------------
FG01898.1 	LGNN-----GIEWFPEAEKDDDFKTNQQ------------KHIQTDEEDSSISQELRRRR
AN6119.1  	TEQDYPGIRNIDWESEYQVGDETPTQESTAFTDESHEEEGKDVTVDTDDEDDDYYDRWEP
          	  :: ..  .*:  .. .  .   ::. :: :..: ..... :  . ..           

MG06906.1 	---------------
NCU01464.1	---------------
FG01898.1 	MRK------------
AN6119.1  	LKWEDQRPLGFRRRR
          	   ... . .
```
